# Supplementary material for: Acceptance and compliance with micronutrient powder (MNP) among children aged 6–23 months in northern Nigeria
Source: PLOS Glob Public Health. 2022 Oct 17;2(10):e0000961. doi: 10.1371/journal.pgph.0000961 (PMC10022258; doi:10.1371/journal.pgph.0000961)
Supplement: S1 Dataset — (ZIP) [file pgph.0000961.s008.zip › S8_Dataset/In-depth Interviews.zip!/In-depth Interviews/110715_K_DW_BZ_2_CG.pdf]

PHASE TWO (2) HOME-FEEDING TRIAL IN-DEPTH INTERVIEW WITH A MALE CAREGIVER AT RAFIN GORA COMMUNITY, DANKO WASAGU LOCAL GOVERNMENT AREA OF KEBBI STATE

ON 11st JULY, 2015

I: please I want you to describe people in your immediate family living in your house hold?

R: Did you mean all of my immediate family living in my house**.**

I: Yes, I mean your immediate family.

R: My name is Bala I have four daughters that is from the female side and three boys that is from the male side. The one under 2years that is 15 months her name is Adama, she is the one that receive the MNP.

I: We have heard of food challenges for young children in this setting related to the seasons. What mechanism could be adopted in coping these food challenges?

R: I don't have much problem on feeding of our children because no matter how, you most look for what children is going to eat or otherwise they won't aloud you to seep meaning that they will crying the whole night.

I. uh

R. The food we gave them are: *towo, shinkefa, kunu*.

I: I mean, we have heard of food challenges from your community here recently, we discovered that some foods are seasonal?

R: During rainy season we cultivated millet, sorghum, rice, maize, cowpea that is beans, ground nut, soya beans, etc. During dry season we okra, sugarcane, tomatoes, spinach, amaranths, *zogala*, pepper, sugar cane, and even dry season rice, etc. Ones there is no enough food for the mother you will gate to know that the child too is affected because as you sow a sign of hunger from the mother you will definitely find the sign of hunger from the child.

I: What mechanisms could be adopted in resist these challenges?

R: Mechanisms to cope the challenges is that we try as much as we could to store the harvested produce in order to be available all year round.

I: One thing we heard in other interviews that caregivers often give their children 6-23 months is "tea". Could you please explain "tea" for me?

R: We use buy *shayi* outside the house which contain different ingredients such as boil water milk, lipton, ginger and other sugar and sometimes bounvita. We normally drink it with *burodi,* ether in the evening time and or in the morning time.

I: Thank you for that explanation. Now I want us to talk about the MNP that you received. Could you tell me about your experience with the product?

R: Yes, i am not around when they gave my wife the MNP product, the health personnel that gave the product to wife she mate my daughter suffering from Malaria and she said we should not start giving her the MNP until she gate well.

I: OK. I want you to talk more about the positive aspects of the experience with MNP.

R: My experience on MNP product is very good for every child to be given the MNP product because it is very good for their health, making the child very active, she is now stronger and healthier, she eat well, in fact she is looking fresh now.

I: Please I want you to tell me a specific story relating to improvement in your child's health compared to before using the MNP.

R: My first child suffers from Tamowa that is *Rashinjini* (anaemia) we went to the clinic for medical check up the doctor told us that our child is suffering from Tamowa he gave us some medicine and he referred us to the clinic close to our own village where we started collecting Tamowa Food. We use to go for check up from time to time and continuous collecting the food for tamowa. Two months letter if see the way she is playing, running up and down you get to know that she is now stronger and healthier but, before the she looking inactive, she don’t even take *kunu,tuwo, shinkafa,* etc, even she does she still look emaciated but, now she take everything you gave to her too much.

I: Thanks you for your MNP story you gave to me. Could you talk about any general challenges you had during the MNP trial?

R: My daughter was seek you brought your MNP product, the health personnel that gave the product to my wife she mate my daughter suffering from Malaria and she said we should not start giving her the MNP until she gate well.

I: Tell me about any specific barriers that made it hard to use the MNP appropriately

R: Honestly, if there is any challenge, I am yet to notice any, which means there is no any barrier.

I: Ok. Some caregivers mentioned that it was hard to store the MNP without it being taken by others or eaten by rodents. Could you please talk about your experience?

R: In our case we don't have any problem with our storage, we don't encounter anything like that and then we are not hoping that to happen.

I: Do you observed any side effect of the MNP on your child?

R: I did not observe any side effect on her regarding the MNP product.

I: Some mothers indicated that it might be hard to remember to use MNP. Did you have a similar experience?

R: I don't have any problem like that because even if she forget to gave her the MNP product in the morning when she remembered in the afternoon or in the evening she will gave her. I normally use to ask her in order to remind her that if she did not forget to gave the MNP because we are human being, we normally forget thing some time but in the case of MNP I know she is really trying, because since the date she started administering the MNP she never forget to gave her the NMP.

I: Thanks for the information. What specific solutions could you suggest to address each of the barriers or challenges mentioned above?

R: Initially I told you that we did not encounter any problem with the MNP Product.

I: Thanks you, Let us now talk about the child's feeding and response to the MNP. Please talk about how you feed the MNP to the child usually.

R: Tell me how can I not feed my baby with MNP, as I told you initially that she is now stronger and healthier and also good looking. If see my child the way he is playing, running up and down you get to know that she is now stronger and healthier but before the child look inactive.

I: So what food do you use to mix with MNP?

R: It better if you can allow me to go and ask my wife because she is the one that is with the child throughout the day.

I: I mean the kind of food she uses to mix with MNP for your child?

R: Ok, *kunu, fura, miya* other liquid food available when giving the MNP to the baby.

I: When do you usually give the MNP to the child?

R: I don’t know the specific time that she giving MNP to our baby but, ask my wife because she is the one that is with the child throughout the day.

I: To what extent does the child likes or dislikes the taste of the MNP?

R: When baby sow you putting something her liquid feed apart from sugar she will not take for you. My wife said whenever she want to mix the MNP with her food she most hide it for her not to see the MNP product.

I: I want to know how, if at all, consumption of MNP impacted the child's diet.

R: The baby food in takes have increased, compared with before he started consuming the MNP product, she reduce suckling the breast milk of his mother, once she take *tuwo, shinkafa,fura, gwabe, kunun* that is enough for her to take time before consuming anything again or suckling the breast milk of her mother.

I: In other communities, we noticed that sharing of the MNP occurred. Could you talk about any sharing of the MNP when you used it?

R: No we never give it to any one apart from child that who's MNP is given for and we never jump any day since the date we started given the MNP to our baby and every day we gave him one sachet of MNP.

I: Please could you tell more on any sharing of the MNP due to food shortage during lean season?

R: Honestly, I have never heard any problem of food shortage in my house but, sometimes due to the negligence of women, if she did not prepare it she ask *kunu* from her friend so that she can mix the MNP with in order to feed the child.

I: Please tell me whether the mother or father do ate out of the MNP as a snack

R: No, by all indication she doesn't ate it. She gave it to the child alone as prescribed one daily.

I: What happens to the leftovers of the MNP?

R: We don't give him more than what he can't finish. His mother told me that there are always no leftovers and I notice it myself. I initially told you that his feed intake increases seriously so even if you gave him what you know he can finish, he will still ask for more.

I: Tell me whether 60 sachets will be enough for 60 days.

R: Yes, we need it even if it's more than 6o sachet.

I: Why did you say they will not be enough?

R: B because you might loos one or two do to our negligence so we need replacement and there is no any additional one to replace, even 60 will be enough but, we are urging if you have any chance of adding another sachets above 60 sachets do as much as possible.

I: Ok. Now that you are using the MNP, could you talk about how you perceive it?

R: The perception I have is that the MNP is very easy to use. Just as the person who brought it explained, he said that it can be administered in the morning, afternoon and evening. But he recommended morning. So that, when one forgets to administer it in the morning, the he can still administer it in the evening time**.**

I: Do you see the MNP as food, medicine or something else?

R: Honestly I see it as medicine.

I: OK. Why?

R: It is because the way you prescribe it to me is almost the same way that the health personnel in the clinic use to prescribe the medicines for us when we are seek and because it helps in improving the health status of the children's.

I: If this product were cheaply on sale in your community, would you purchase it?

R: Yes, I can buy, because it is good for my child, it's making him stronger and healthier. So I am ever ready to buy whatever is good and improvement for his health.

I: How much would you be willing to pay for a sachet?

R: Yes, I can buy it at the rate of 20-30 naira per sachet.

I: You are giving me great information. Now please tell me some ways that you believe would be effective for promoting MNP to caregivers in the community so that they would comply with using it appropriately

R: W hen you want promote the MNP product is by use of the people who already have the product so that you will use them a sample for reference and bringing enough of you MNP product, so that our people will have it for their children, enlightenment campaigns, educating our people on how the MNP product should be given; how it should be use; its importance and positive effect on the children. If possible use our indigenous people to be explaining these product and use of female for their female counter part. If that can be achieved I assured you that the product will reach where you are not even expecting.

I: Are there other ways we can follow to ensure the caregivers use it appropriately?

R: You can only achieve appropriate use of the product by the use of husband or any influential person the house, so that he will be asking and reminding them if the product is been use appropriately or not.

I: Thank you so much on that information. What people most influence your opinions and knowledge about feeding practices?

R: In any community they have their own settings we have our own way of feeding behaviour that is we are expected to do, for example the mother or the father are the one feed the child of under two years or the elder sisters or brothers too can feed the chid. So my religion is the one that influence opinion the feeding practice.

I: Ok. Who influenced your decisions regarding feeding your baby with the MNP?

R: You know I told you that I appreciate the way our village head is handing issues like that because treated everybody equally. He send somebody to call me in my house I mate him discussing MNP issues with person in charge of MNP distribution that is Malama Zainab, they explain everything to me pertaining MNP usage and advantages.

I: Where would be good places to disseminate health information related to MNP?

R: The good place to disseminate health information related to MNP is in our village head palace or the heath facilities because is only easiest way to gather people of this community.

I: Thank you for that explanation. Who in your community do you trust the most for receiving messages about infant and young child feeding?

R: The person I can trust in my community is our village head, because ones we have any problems they are the ones that we will run to, whatever concern this village they worked hand in hand to find the best solution to us and our community.

I: What forms of media do you prefer for receiving MNP messages? Brochure/Flyer/Posters/radio/sms?

R: Yes, I know posters are very good information displayer for any king of information you want to send to us because majority of us cannot read or write but we all understand posters.

I. uh

R. because is something that you can see and sometimes you can get detail information about the MNP.

I: Could you please tell me what ways would you prefer to receive education regarding IYCF and MNP?

R: I initially told you that it should be through the health personnel in this community and though our village head and our religious leader and other influential people in this community.

I: Thanks so much for that suggestions. Tell me about specific examples of other successful health education campaigns in this community.

R: Yes we have successful programs, such as Malaria the health personnel did allots of enlightenment campaign on malaria in which they achieve what they want to achieved, they gave us mosquito net, drugs free of charge and we have to use our own money to buy them when the free once are not available. So they are successful now.

I: What are the best community channels to distribute the product?

R: The right channels to use in distributing the MNP is the village head, because the village head knows everybody in his community and we all know that he is honest and troth. So whatever you gave out to people, I assured you in as far as the village head concern the product will reach where you want the product to reach or intended people for the product.

I: Thanks sir. I want to hear some specific messages you think would effectively promote the product.

R: I have told you earlier that the village head with the help of town announcers.

I: Please could I hear some specific message you think would effectively promote the MNP product?

R: The message should be detail explanation on how it should be used and how important it is for the children.

I: Ok. What other specific messages could be used to ensure that it will be used appropriately?

R: Enlightenment campaigns, educating our people on how the MNP product should be given; how it should be use; its importance and positive effect on the children.

I: Thank you so much for that message. What specific messages could be used to promote its use but limit the sharing of it?

R: The message should be detail explanation on how it should be used and how important it is for the children. You should explain to caregivers that these is the target children for the MNP product and is main for them alone, so that you can limit MNP sharing among caregivers or friends.

I: Thank you so much. Finally, is there anything else you can share about your experience with this product, placement, price?

R: I am ok with the product; the price too is ok for me as i said earlier that 20-30 Naira per sachet. There is no need to change anything from the product, so we are happy and excited about this product.

I: What else would you recommend to develop an effective programme?

R: My recommendation is that you should make use of village heads and religious leaders in introducing anything in this community.

I: Thanks.
